# People with Disabilities in the Workplace: Results of a Survey Conducted among Polish and Finnish Employers

**DOI:** 10.3390/ijerph182010934

**Published:** 2021-10-18

**Authors:** Alicja Grześkowiak, Urszula Załuska, Dorota Kwiatkowska-Ciotucha, Cyprian Kozyra

**Affiliations:** 1 Department of Econometrics and Operational Research, Wroclaw University of Economics and Business, 53-345 Wrocław, Poland; alicja.grzeskowiak@ue.wroc.pl; 2 Department of Logistics, Wroclaw University of Economics and Business, 53-345 Wrocław, Poland; urszula.zaluska@ue.wroc.pl; 3 Department of Statistics, Wroclaw University of Economics and Business, 53-345 Wrocław, Poland; cyprian.kozyra@ue.wroc.pl

**Keywords:** disability, inclusive employment, quantitative research, employers’ opinions

## Abstract

The key aspect of the inclusion of people with disabilities (PwD) in the workplace is how they are perceived by employers who make decisions on hiring employees. The article presents the results of CAWI (Computer Assisted Web Interview) research conducted among Polish and Finnish employers (*n* = 414) in 2021 using a proprietary questionnaire. Employers were asked to assess the state policy in the field of PwD’s inclusion, the social atmosphere in this respect, the level of acceptance of privileges/special solutions dedicated to PwD in the workplace and the knowledge of the specificity of disability. When analysing the obtained data, we verified the differentiation of Polish and Finnish employers’ assessments, the impact of respondents’ characteristics on their assessment and the relations between the assessments of various aspects of PwD’s inclusion. For the analysis, we used the *t*-test of independent samples for equality of means and the Pearson correlation coefficient. The results showed that Finnish respondents assess the conditions for the full inclusion of PwD much better than Polish ones. The characteristics most differentiating employers’ assessments is gender and the fact of employing PwD. There were also correlations between the responses of respondents in both countries to three out of four analysed questions from the questionnaire. The differences found in this study indicate that it would be worth extending the research to other European countries to generalize conclusions about the influence of cultural determinants on the situation of PwD on the labour market.

## 1. Introduction

The last decade has seen growing openness and acceptance of people with disabilities both in public space and in the workplace. This has been shown by formal and legal solutions, including the ratification of the United Nations Convention on the Rights of Persons with Disabilities [1] by most countries. The slow improvement of the situation has been indicated by the statistical data showing an increase in the percentage of employed PwD [2,3]. Percentage of persons with disabilities in employment in 2020 was 54% in Finland and 42% in Poland, employment gap between persons with disabilities and persons without disabilities was 20 pp. in Finland and 32 pp. in Poland. The results of semiotic research carried out on the basis of the analysis of cultural texts, i.e., texts from the press or Internet, films or advertisements, also indicate the improvement of the image of people with disabilities in the media and the willingness to introduce “normality” to the perception of this issue [4]. However, the way to the full inclusion of PwD is still very long. Despite the increasing employment rates of people with disabilities in various countries, their levels are still lower than those of people who do not suffer from any disabilities [5,6,7].

**Figure 1:**
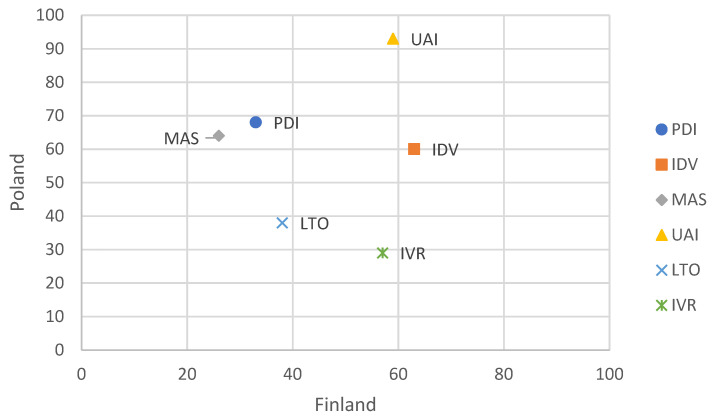
The values of indexes of cultural dimensions according to Hofstede [17] for Poland and Finland.

The results of the research conducted by many teams of scientists indicate that the low level of knowledge about disability is one of the main causes of unfavourable social perception of PwD, and consequently a barrier to their effective inclusion [8,9,10]. The key aspect of the inclusion of PwD in the workplace is how they are perceived by employers. It is because the latter make ultimate decisions on who gets a job [11,12,13]. However, the perception of people with disabilities depends on the conditions present in a given country, that is, applicable legal solutions, support system, social atmosphere, state policy in this respect or knowledge of the subject. The situation may look different in different countries because the perception of PwD may be significantly influenced by the specific features of the culture of a given place, measured, e.g., by means of the so-called dimensions of culture. Hofstede proposed standardized quantitative indexes on a 0–100 scale measuring particular dimensions of culture, which can be used to describe the characteristics of communities in various nationalities [14,15,16]. In the research, the results of which are presented in this article, we wanted to check how employers from countries characterized by different cultural dimensions perceive issues of PwD, especially in the workplace. Poland and Finland were chosen for comparisons as they vary substantially in terms of the cultural dimensions proposed by Hofstede (cf. Figure 1).

What seems to be important from the point of view of disability issues are the indexes measuring three dimensions, namely PDI (Power Distance), MAS (Masculinity) and UAI (Uncertainty Avoidance). The PDI index groups communities according to the power distance—the higher the PDI value (higher power distance), the greater the acceptance of social inequalities resulting from the privileges of power. The dimension of femininity vs. the masculinity of society is described by the MAS index. Feminine societies (low MAS index) are characterized by strong care for relationships with others, in contrast to masculine societies (high MAS index) where the emphasis is put on competition and achieving success. The level of risk in ambiguous or unfamiliar situations experienced in a given society is measured by the UAI index, the high value of which indicates high social concerns about new situations or unknown behaviours. According to Hofstede’s dimensions, Poland is classified as one of the countries with high power distance, characterised by masculine culture, with a high value of the uncertainty avoidance index. On the other hand, Finland is classified as one of the countries with low power distance, with a typically feminine culture and relatively high openness to new and unconventional behaviours and situations. The concept and results of Hofstede’s research have both supporters and opponents (e.g., [18,19,20]), whereas criticism concerns substantive as well as methodological issues. However, it is worth noting that research done by other authors confirms the cultural diversity of European countries, especially in the context of social relations, including openness to people with disabilities. The advantage of Hofstede’s approach is the quantitative nature of the research results—this makes it possible not only to determine the differences between the dimensions of culture in different countries but also to assess their size. The following research hypothesis was formulated: In Poland and Finland, countries characterized by different cultural dimensions, opinions of the employers on issues concerning PwD vary substantially.

## 2. Materials and Methods

### 2.1. Data Collection Process

The comparative research was conducted using the CAWI method (Computer-Assisted Web Interview) in the period of February–March 2021 on samples of employers from Poland and Finland by an entity that specializes in social research. Employer was understood as a decision-maker in the field of hiring employees in a given organization, e.g., an owner, a managing director or the head of the HR department. According to the assumptions, the minimum sample from each country was 200 people and was controlled in terms of the size of entity. The final structure of the companies covered by the research was consistent with the distribution of companies of various sizes in each country. The research covered respondents from enterprises employing at least 5 employees. They were recruited mainly from online panels available in a given country, and the sample was also partially obtained from invitations from company databases. The respondents represented companies from different branches that were defined according to the first level of the NACE classification (Nomenclature statistique des Activités économiques dans la Communauté Européenne). One interview lasted about 12 min and covered two areas. The first one referred to preparation of employees for current and future challenges, i.e., the level of various competences. The second focused on conditions for the full inclusion of PwD in a given country as well as openness to employing PwD. In the part concerning PwD, the respondents were asked about the preparation of employers for employment of PwD, the state policy in this area and the degree of acceptance of employee privileges/special solutions for PwD.

The data were collected by the proprietary questionnaire (Table 1) by using a four-point Likert scale and scored from 1 (“definitely not”) to 4 (“definitely yes”). The analysis of the answers made it possible to assess the conditions ensuring or hindering the full inclusion of people with disabilities, especially in the workplace.

The research questionnaire received a positive opinion from the ethics committee of the Wroclaw University of Economics and Business. Participation in the study was voluntary. Respondents were guaranteed the confidentiality of their responses in accordance with the applicable data protection law (GDPR) and the code of ethics in market and public opinion research (ESOMAR). They were also assured that the survey results would be analyzed only in form of aggregated statistics.

### 2.2. Analytical Methods

In order to check the possible differentiation in the perception of the issue of disability by Polish and Finnish employers, three research questions were formulated:

The first research question: Do the national conditions for the full inclusion of people with disabilities in the workplace differ according to Polish and Finnish employers?

The second research question: Do the characteristics of respondents influence the assessment of these conditions and are there any differences in this respect between the opinions of employers from Poland and Finland?

The third research question: Are the assessments of different aspects of inclusion of people with disabilities in the workplace related to each other?

Various methods of data analysis were used to obtain answers to the research questions. In order to evaluate whether the responses of respondents from both countries/due to different characteristics were significantly different, the *t*-test of independent samples for equality of means was used. The *t*-test was preceded by Levene’s test to assess the equality of variance in the samples (cf. [21]). If the hypothesis of equality of variance in Levene’s test was rejected, the *t*-test was carried out using the approach *equal variances not assumed*. In other cases (no reason to reject the equality of variance hypothesis), the *t*-test was performed using the option of *equal variances assumed*.

The answer to the third research question was possible owing to the analysis of correlations between the answers given by individual respondents to the questions from the proprietary questionnaire presented in Table 1. In order to evaluate the strength of those correlations, we relied on the Pearson correlation coefficient. Additionally, in order to identify groups of similarly shaped variables, we used a visualisation based on an ordered correlation matrix [22]. The variables were grouped using a hierarchical agglomeration procedure.

For statistical calculations and visualisation, we used the IBM SPSS 25.0 statistical package, the R programme and also functions of the MS Excel 2019.

As for the questions that contained more than two variants of answers, for the needs of further research and the research methods used, the answers were recoded to two categories. This was the case for all questions except for gender characteristic. In the research, the respondents gave their age in years, and it was decided to distinguish a group of people up to 35 years old and older. This threshold resulted, on the one hand, from the situation on the labour market (a period of gaining a position vs. period of stability), and on the other hand, from the percentage distribution for the sample of respondents (more or less 50/50). The characteristic concerning the knowledge of the issues of PwD was originally measured on a four-point scale (from “none” to “very good”). After aggregation, we distinguished a group of people declaring a “very good” or a “good” level of knowledge of this issue and a group of remaining respondents. The division used was justified by the previous analyses carried out by the research team as part of the former research. As far as the company’s size was concerned, we distinguished micro and other companies. It was mainly related to the fact that employees from the smallest companies had to perform various work, often in different areas. When it comes to the question about employing PwD, the analyses did not include the respondents who indicated a lack of knowledge in this field. The last two characteristics included in the research concerned opinions on the importance of specific competences that may affect the perception of PwD in the workplace. The respondents used a 10-point scale, from 1 to 10, where 1 meant “a completely meaningless competence” and 10—“a competence of key importance”. It was assumed that the answers from 8 to 10 testify to the great importance of a given competence according to employers, while the remaining answers—to its lesser importance.

## 3. Results

### 3.1. Sample Characteristics

As part of the CAWI research, we collected 415 complete questionnaires from employers. The qualitative assessment of responses indicated the necessity to eliminate a questionnaire from one of the interviews due to the discriminatory nature of the opinions given in the open-ended question and the lack of consistency in the responses to closed-ended questions. Ultimately, 200 questionnaires from Finland and 214 questionnaires from Poland were included in the analyses. The characteristics of the research sample due to the features analysed in the article are presented in Table 2. The respondents represented companies of various sizes of staff, operating in various sectors of the economy. In terms of gender, slightly more respondents were men. It is worth noting that significant differences were observed between the countries as the Polish sample was dominated by women, whereas the Finnish one—by men. The sample was diverse in terms of age—the youngest respondent was 20 years old, whereas the oldest one—74. The average percentage of companies employing PwD was 33.6—significantly fewer companies employ PwD in Poland than in Finland (a difference of 10 percentage points). Nearly 1/3 of the respondents described their knowledge about disability as good or very good.

### 3.2. Evaluation of Differences in Opinions Expressed by Respondents in Finland and Poland

**Figure 2:**
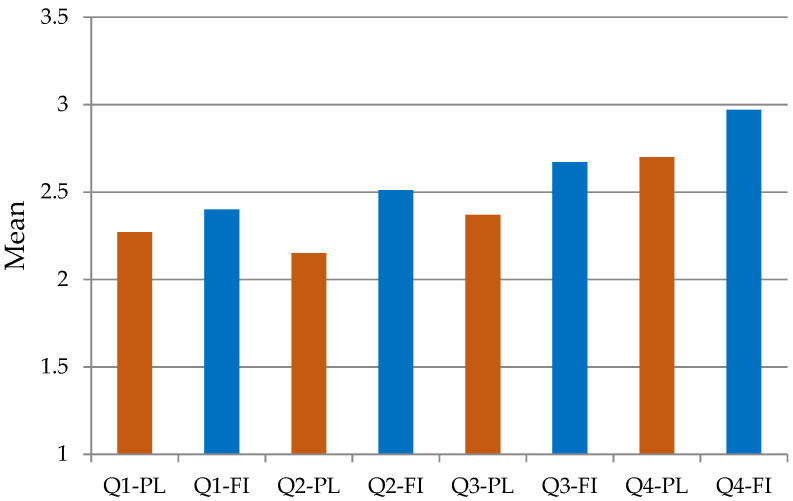
Average responses to questions Q1–Q4 in Finland (FI) and Poland (PL).

The situation in Finland and Poland with regard to issues concerning PwD included in questions Q1–Q4 was evaluated by the respondents from both countries slightly differently. Figure 2 represents the mean average of answers of the employers by country. The mean value of the Q1–Q4 answers given by the Finnish respondents was higher than the Polish. The highest values were noted for the question about privileges for PwD (means: Finland 2.97, Poland 2.70). The lowest values were observed in Finland for the question concerning employers’ knowledge of employing and organizing work for PwD (mean: 2.40), whereas in Poland—for the question about the state policy (mean: 2.15).

In order to evaluate whether the responses of respondents from both countries differed significantly, the *t*-test for equality of means was used, and its results are presented in Table 3.

It is possible to notice significant differences in the answers to questions Q2–Q4. The respondents from Finland evaluated the effectiveness of the inclusion policy for PwD implemented by the state in a more positive manner (means: Finland 2.51, Poland 2.15) and found the social atmosphere more PwD-friendly (means: Finland 2.67, Poland 2.37). A significant difference in opinions also concerned the acceptance of privileges dedicated to PwD (means: Finland 2.97, Poland 2.70). No significant difference was found only for question Q1 about the knowledge of employing and organising work for PwD (average: Finland 2.40, Poland 2.27). The obtained results indicate that the respondents from Finland evaluate many key aspects of PwD in the labour market in a more positive way.

### 3.3. Evaluation of Differences in Opinions with Respect to Various Covariates

The respondents participating in the research did not form a homogeneous group, hence the further analysis focused on the assessment of differences in the answers to questions Q1–Q4 in relation to various covariates, i.e., gender, age, company’s size, declared knowledge about disability, employment of PwD, opinions on the importance of competences such as working in a varied team and sensitivity to social issues. For each covariate, *t*-tests for equality of means were conducted. The tests were done separately on the basis of the results obtained in Finland and Poland.

Table 4 presents the test results for the question concerning the knowledge about employing and organising work for PwD (Q1). In both countries, significant differences were found in the responses given by men and women, and they followed the same direction—men evaluate the level of knowledge of employers better than women. In Finland, there were some differences in the opinions given by respondents whose companies employed and did not employ PwD. Namely, the representatives of companies that hired PwD better evaluated the knowledge of employers in this field. In Poland, the differentiating characteristic was the knowledge of disability—people who claimed that they were aware of PwD’s issues better perceive employers’ preparation for employing and maintaining the employment of PwD. The tests did not show any differences in terms of age, company’s size and the perception of the importance of the competences indicated in the research in either country.

Table 5 presents the test results for the question evaluating the conduct of an effective policy enabling full inclusion of PwD (Q2). Both in Finland and in Poland, there were significant differences depending on whether or not the company employs PwD. The respondents who have PwD in their workplace evaluate the effectiveness of the state policy in terms of the inclusion of PwD in a more positive manner. In Poland, age and knowledge of PwD issues were also important—younger people gave higher average assessments, and the knowledge about disability also contributed to a better perception of the activities conducted by the state. In Finland, on the other hand, we noted significant differences in terms of gender—assessments given by men exceeded those given by women.

Table 6 presents the test results for the question relating to the social atmosphere of understanding the needs and possibilities of PwD (Q3). In Finland, significant differences can be noticed in terms of two covariates (knowledge about disability, employment of PwD), whereas in Poland only due to one (gender). The Finnish respondents from companies employing PwD have a better perception of the social atmosphere regarding disability than people from enterprises where PwD do not work. A more favourable assessment in Finland is also fostered by declaring the knowledge of a disability. In Poland, the only statistically significant difference occurred in the opinions given by the representatives of different genders. Namely, women were more critical of the understanding of the needs and prospects of PwD showed by society. It is worth noting that for most covariates (age, company’s size, work in a varied team, sensitivity to social issues) there was no reason to reject the equality of means hypothesis in either country.

Table 7 presents the test results concerning the question of the legitimacy of employee privileges for PwD (Q4). In each country, the results for only one covariate appeared to be significant. In the case of Finland, this is an evaluation of the importance of sensitivity to social issues. People who assessed the importance of this competence as high approve of special privileges for PwD to a greater extent. In the case of Poland, the age of the respondents turned out to be significant—older people are more willing to accept specific privileges for PwD, e.g., shorter working days or additional leaves. It is worth emphasising that for this question the number of significant differences found for the analysed covariates was the lowest.

Summarising the results concerning the differentiation of assessments due to different covariates, it should be emphasised that there is no uniform pattern for all questions. However, it is possible to indicate some regularities:

− If significant differences were found in terms of gender, it meant a lower evaluation given by women than by men (Q1—Finland and Poland, Q2—Finland, Q3—Poland),
− If the knowledge of disability was a significantly differentiating characteristic, the respondents familiar with the issues of PwD gave higher average assessments (Q1—Poland, Q2—Poland, Q3—Finland),
− If employing PwD in the respondent’s company was a significantly differentiating characteristic, the affirmative answers were accompanied by averagely higher assessments (Q1—Finland, Q2—Finland and Poland, Q3—Finland),
− Age was an important characteristic only in Poland for two questions and its significance varied—younger people evaluated the state policy better (Q2) but at the same time they were more often against privileges for PwD (Q4),
− No average assessments in Q1–Q4 showed any significant differentiation in terms of company’s size,
− No average assessments in Q1–Q4 showed any significant differentiation in terms of the importance of a competence defined as the ability to work in a varied team.

### 3.4. Correlations of Answers to Questions Q1–Q4

The subject scope of questions Q1–Q4, including the context of PwD functioning in society, prompts us to check whether there are any relations between the assessments of the issues under consideration. The evaluation of relationships was performed with the use of correlation analysis. The numerical results along with the assessment of significance are presented in Table 8.

The values of the Pearson correlation coefficients varied. The answers to the first three questions (Q1–Q3) are pairwise significantly correlated (*p* < 0.01) in Finland and Poland. The weakest correlations were found between the answers concerning privileges for PwD (Q4) and the other questions. In the case of Finland, all correlations between Q4 and other variables are statistically insignificant, whereas in Poland the insignificant correlation is the one with the variable representing the social atmosphere (Q3). The correlation coefficients between other variables (Q1 and Q2) are low, but statistically significant (*p* < 0.05).

**Figure 3:**
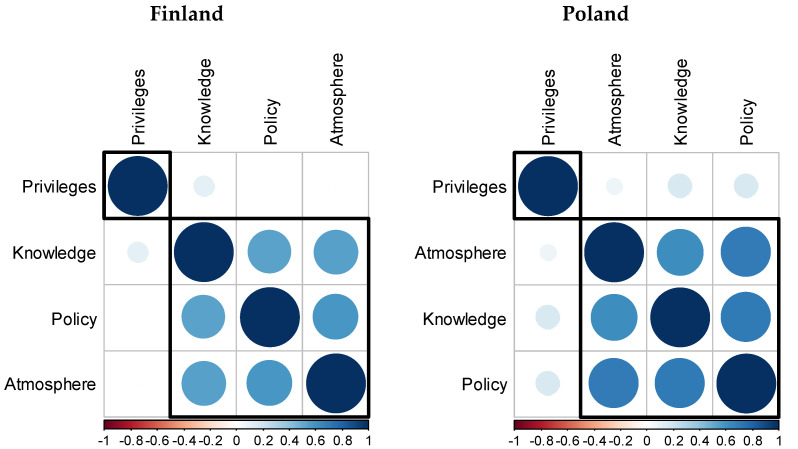
Visualization of ordered correlation matrixes for questions Q1–Q4.

In order to synthetically present correlations between the answers to questions Q1–Q4 and to identify groups of related variables, we relied on the visualisations of the ordered correlation matrix using the corrplot package of the R programme (Figure 3). The presented visualisation reflects the direction of the correlation (represented by a different colour—red and blue) and its strength (represented by the colour intensity and the size of the circles). The variables were grouped using a hierarchical agglomeration procedure and two separate groups were distinguished.

The representation in Figure 3 shows the identified regularities. Correlations between the answers to questions Q1–Q4 are positive and of different strength. Both in the case of Finland and Poland, the applied clustering procedure distinguished two clusters. One contained variables strongly correlated with each other, i.e., Q1, Q2 and Q3, whereas variable Q4 representing privileges is isolated from the others due to the lack of a strong correlation with them. The assessments of respondents from Finland and Poland concerning the knowledge of employers, the inclusion policy carried out by the state and the social atmosphere conducive to the inclusion of PwD are relatively strongly and positively correlated with each other. The evaluation of the legitimacy of privileges is not closely correlated with the assessment of other aspects.

## 4. Discussion

The comparative research conducted on representative samples of Polish and Finnish employers on the perception of determinants in the area of inclusion of PwD gave interesting results. The research subjects were the answers of respondents assessing the level of knowledge about disability displayed by employers, the state policy in the area of PwD’s inclusion, the social atmosphere in this regard, and support for privileges/special solutions for PwD in the workplace. The study had an innovative character. The research on the perception of disability in the workplace on representative samples of employers from two culturally different countries should be considered unique. The obtained results should contribute to the development of effective solutions for PwD inclusion in the workplace. When analysing the results at the country level, it was possible to notice that in Finland higher mean values were obtained for all questions, with statistically significant differences obtained for three out of four analysed questions (except for the level of employers’ knowledge). It means that the Finnish respondents evaluated the conditions in their country enabling the full inclusion of PwD in a far more positive way than the Polish ones. This confirms the potential impact of cultural determinants on openness towards people with disabilities not only in the area of the social atmosphere of understanding the needs and possibilities of this group of people or special employee privileges but also in terms of political conditions conducive to their full integration. The compared countries are characterised by different values of indexes for selected dimensions of culture according to Hofstede. Relatively low mean values in both countries concerning the level of employers’ knowledge about disability indicate that there is still a lot of space for undertaking activities in the field of informing people about these issues, e.g., in the form of social campaigns. The lowest mean value obtained for the Polish respondents in the field of state policy indicates that they are critical of the current activities in this area (mean: 2.15 on a four-point scale).

Table 9 presents the summary of the obtained significant differences of opinions in the answers to questions Q1–Q4 in Finland and Poland in terms of the characteristics of the respondents included in the research. It is worth emphasizing that for most of the assessments given in both countries, no differences in the perception of the determinants of full inclusion of PwD in the workplace were noted due to the respondents’ characteristics. This was the case in both countries for 21 results out of 28 differences checked. Similarities for both countries in the differentiation of opinions occurred in two cases: the assessment of employers’ knowledge (in both cases, men evaluated the level of employers’ knowledge higher) and the assessment of state policy (those who employ PwD gave more positive evaluations). Taking into consideration the obtained differences for both countries, most of them occurred for the assessment of state policy (five in total—two characteristics in Finland and three in Poland). In total, the most differentiating characteristics in both countries were gender and the fact of employing people with disabilities (four indications each—two for gender in both countries, three for the question about employing PwD in Finland and one for Poland). In Finland, the most differentiating characteristic was the fact of employing PwD (for this feature, three significant differences were obtained for the four questions asked), followed by gender (two significant differences for four questions). In Poland, two significant differences were noted for characteristics such as gender, age and knowledge about disability. Interestingly, the importance of the competence concerning sensitivity to social issues confronted with the question about privileges for PwD in the workplace revealed significant differences only in Finland (higher acceptance of privileges in the case of a higher evaluation of importance of this competence). On the other hand, the opinions differed significantly in terms of the distinguished age groups only in Poland. This concerned questions about state policy (younger respondents evaluated it better) and privileges/special solutions for PwD in the workplace (older respondents expressed greater acceptance). It is also worth noting that we did not observe any differences in either of these countries in the opinions of employers due to company’s size and the assessment of the importance of competences of working in a varied team.

The obtained results confirm the previous ones obtained by other research teams conducting research on openness to the needs of PwD. The perception of disability is conditioned by knowledge and experience in this area, and the research confirms a positive correlation between favourable attitudes towards people with disabilities and having experience in this field [9,11,23,24,25,26,27,28,29,30]. On the other hand, women are more open to people with disabilities than men, but at the same time they are more critical of external conditions, including systemic solutions, the social atmosphere or the level of knowledge about disability [4,31,32].

When answering the third research question, it should be stated that there is a correlation between the respondents’ answers to three out of four analysed questions from the questionnaire in both countries. This correlation is positive, which means that a better assessment of the effectiveness of state policy enabling full inclusion of PwD is accompanied by a higher assessment of the social atmosphere of understanding the needs and possibilities of PwD and the knowledge of employers about employing and organising work for this group of people. However, opinions in the above scope do not correspond to the assessment of the need for special employee privileges for PwD. In this case, the values of correlation coefficients with other analysed issues indicated a positive correlation, but they had low values, and the hierarchical agglomeration procedure applied distinguished the answers to this question as separate from the others. The authors of the article obtained similar results in previous research dedicated to the perception of PwD that was conducted in eight different European countries on a sample of over 4000 respondents (analyses in this area have not been published yet). In the case of these analyses, even a negative correlation was found between the assessment of the determinants of inclusion of PwD and the assessment of the need for special employee privileges at the level of mean assessments from the countries covered by the research.

## 5. Conclusions

The analyses carried out on representative samples of employers from Poland and Finland allowed us to confirm the research hypothesis, as they showed the occurrence of significant differences in the perception of disability, which may indicate an influence of cultural differences on the evaluation of the situation in this area. To our best knowledge, so far, there has been no research on openness towards people with disabilities in the workplace in Poland and Finland that could be comparable with results presented in this article. Due to that, it is impossible to directly relate the obtained results to the results of other researchers. The research on the functioning of people with disabilities in society indicates significant disproportions between different countries in various dimensions [33,34], which motivated the authors to conduct their own comparative research that was narrowed down to the assessment of the situation of people with disabilities in the workplace. This subject has been analysed by other authors but in different contexts. Researchers focused on the inclusion of people with disabilities in the workplace in specific countries [35,36,37,38,39], describing activities related to inclusion [40,41] and presenting various concepts of social inclusion/social exclusion of PwD [42]. Summarizing this research, however, it is necessary to point out its limitations. First of all, it was conducted in only two countries, which significantly limits the possibility of drawing conclusions. In the future, it would be worth extending this type of research to other countries as well. Secondly, it is worth analysing the selected questions not only in relation to the characteristics of respondents participating in the survey, but also other issues raised in the questionnaire, such as acceptance and willingness to employ people with various types of disability or openness to training and improving competences. Thirdly, other analytical methods may be applied including multivariate approaches, e.g., a regression analysis with a dummy variable representing countries and with covariates as controls. Last but not least, it would be worth comparing the opinions of research participants with official statistics in the area of professional activity of people with disabilities.

## Figures and Tables

**Table 1:** Questions from proprietary questionnaire.

No	Question
Q1	How do you think employers in your country get sufficient knowledge on how to employ a person with disabilities and organize his/her work?
Q2	In your opinion, does your country carry out an effective policy that allows for full integration of the people with disabilities?
Q3	In your opinion, is there social atmosphere of understanding the needs and possibilities of people with disabilities in your country?
Q4	Do you think that the people with disabilities who have a job should have special employee privileges, for example, a shorter working day, longer holidays, etc., in your country?

**Table 2:** General characteristics and percentage distribution of the study participants.

Characteristic	Categories	Percentage of Respondents (*n* = 414)		
		Whole Sample	Finland	Poland
Gender	Female	49.3	36.5	61.2
	Male	50.7	63.5	38.8
Age	Up to 35 years old	49.8	50.5	49.1
	Over 35 years old	50.2	49.5	50.9
Knowledge about	Good or very good	35.7	31.8	39.3
disability	Average or none	64.3	68.2	60.7
Company’s size	Up to 9 people	39.4	40.5	38.3
	10 and more people	60.6	59.5	61.7
Employment of PwD	Employs	33.6	38.8	28.8
	Does not employ	66.4	61.2	71.2
Assessment of competence: working in a diverse team	Very important	49	49.5	48.6
	Another assessment	51	50.5	51.4
Assessment of competence: sensitivity to social issues	Very important	39.1	40.5	37.9
	Another assessment	60.9	59.5	62.1

**Table 3:** Comparison of mean answers to questions Q1–Q4 in Finland and Poland (*t*-test of independent samples).

Question	*t*-Test Score	*p*-Value
Q1—Knowledge	−1.590	0.113
Q2—Policy	−4.608	0.000
Q3—Atmosphere	−3.863	0.000
Q4—Privileges	−3.749	0.000

**Table 4:** Results of *t*-tests for equality of means of answers to question Q1 (Knowledge) according to covariates.

Covariate	Finland		Poland	
	*t*-Test Score	*p*-Value	*t*-Test Score	*p*-Value
Gender	−1.975	0.050	−2.720	0.007
Age	1.114	0.267	0.596	0.552
Company’s size	0.617	0.538	−0.385	0.701
Knowledge about disability	−1.927	0.057	−2.224	0.028
Employment of PwD	3.052	0.003	0.709	0.479
Work in a varied team	1.797	0.074	0.031	0.975
Sensitivity to social issues	0.755	0.451	−0.355	0.723

**Table 5:** Results of *t*-tests for equality of means of answers to question Q2 (Policy) according to covariates.

Covariate	Finland		Poland	
	*t*-Test Score	*p*-Value	*t*-Test Score	*p*-Value
Gender	−2.961	0.003	−1.266	0.207
Age	1.565	0.119	2.744	0.007
Company’s size	0.689	0.491	0.062	0.950
Knowledge about disability	−1.917	0.057	−3.089	0.002
Employment of PwD	2.529	0.012	2.082	0.040
Work in a varied team	0.640	0.523	0.348	0.728
Sensitivity to social issues	0.806	0.421	0.086	0.931

**Table 6:** Results of *t*-tests for equality of means of answers to question Q3 (Atmosphere) according to covariates.

Covariate	Finland		Poland	
	*t*-Test Score	*p*-Value	*t*-Test Score	*p*-Value
Gender	−1.612	0.109	−2.278	0.024
Age	1.449	0.149	1.056	0.292
Company’s size	0.305	0.761	−0.123	0.902
Knowledge about disability	−2.319	0.022	−1.127	0.261
Employment of PwD	2.468	0.015	0.974	0.333
Work in a varied team	0.230	0.818	1.637	0.103
Sensitivity to social issues	0.400	0.689	1.381	0.169

**Table 7:** Results of *t*-tests for equality of means of answers to question Q4 (Privileges) according to covariates.

Covariate	Finland		Poland	
	*t*-Test Score	*p*-Value	*t*-Test Score	*p*-Value
Gender	−0.357	0.721	−0.985	0.326
Age	−0.946	0.345	−2.441	0.015
Company’s size	0.083	0.934	1.503	0.134
Knowledge about disability	0.132	0.895	−0.456	0.649
Employment of PwD	1.391	0.166	−0.119	0.905
Work in a varied team	−1.716	0.088	−0.661	0.510
Sensitivity to social issues	−3.222	0.001	0.455	0.650

**Table 8:** Correlations of answers to questions Q1–Q4.

	**Finland**			
	Q1—Knowledge	Q2—Policy	Q3—Atmosphere	Q4—Privileges
Q1—Knowledge	1			
Q2—Policy	0.538 **	1		
Q3—Atmosphere	0.547 **	0.590 **	1	
Q4—Privileges	0.116	−0.008	0.008	1
	**Poland**			
	Q1—Knowledge	Q2—Policy	Q3—Atmosphere	Q4—Privileges
Q1—Knowledge	1			
Q2—Policy	0.703 **	1		
Q3—Atmosphere	0.624 **	0.700 **	1	
Q4—Privileges	0.169 *	0.167 *	0.071	1

* *p* < 0.05, ** *p* < 0.01.

**Table 9:** Significant differentiation of opinions in answers to questions Q1–Q4 in Finland and Poland due to the characteristics of respondents included in the research.

Covariate	Finland				Poland			
	Q1	Q2	Q3	Q4	Q1	Q2	Q3	Q4
Gender	X	X			X		X	
Age						X		X
Company’s size								
Knowledge about disability			X		X	X		
Employment of PwD	X	X	X			X		
Work in a varied team								
Sensitivity to social issues				X				

## Data Availability

The dataset presented in the study is available upon request from the corresponding author.
